# Statistical optimization of P(3HB-*co*-3HHx) copolymers production by *Cupriavidus necator* PHB^−^4/pBBR_CnPro-*phaC*_*Rp*_ and its properties characterization

**DOI:** 10.1038/s41598-023-36180-7

**Published:** 2023-06-02

**Authors:** Chanaporn Trakunjae, Antika Boondaeng, Waraporn Apiwatanapiwat, Phornphimon Janchai, Soon Zher Neoh, Kumar Sudesh, Pilanee Vaithanomsat

**Affiliations:** 1 Kasetsart Agricultural and Agro-Industrial Product Improvement Institute (KAPI), Kasetsart University, Bangkok, 10900 Thailand; 2 Ecobiomaterial Research Laboratory, School of Biological Sciences, Universiti Sains Malaysia USM, 11800 Penang, Malaysia

**Keywords:** Biopolymers, Environmental impact

## Abstract

Poly(3-hydroxybutyrate-*co*-3-hydroxyhexanoate) [P(3HB-*co*-3HHx)] is a bacterial copolymer in the polyhydroxyalkanoates (PHAs) family, a next-generation bioplastic. Our research team recently engineered a newly P(3HB-*co*-3HHx)-producing bacterial strain, *Cupriavidus necator* PHB^−^4/pBBR_CnPro-*phaC*_*Rp*_. This strain can produce P(3HB-*co*-2 mol% 3HHx) using crude palm kernel oil (CPKO) as a sole carbon substrate. However, the improvement of P(3HB-*co-*3HHx) copolymer production by this strain has not been studied so far. Thus, this study aims to enhance the production of P(3HB-*co-*3HHx) copolymers containing higher 3HHx monomer compositions using response surface methodology (RSM). Three significant factors for P(3HB-*co*-3HHx) copolymers production, i.e., CPKO concentration, sodium hexanoate concentration, and cultivation time, were studied in the flask scale. As a result, a maximum of 3.6 ± 0.4 g/L of P(3HB-*co*-3HHx) with 4 mol% 3HHx compositions was obtained using the RSM optimized condition. Likewise, the higher 3HHx monomer composition (5 mol%) was obtained when scaling up the fermentation in a 10L-stirrer bioreactor. Furthermore, the produced polymer's properties were similar to marketable P(3HB-*co*-3HHx), making this polymer suitable for a wide range of applications.

## Introduction

Plastic pollution has emerged as one of the most critical global environmental challenges. Evidently, the COVID-19 pandemic has contributed to an enormous surge of petroleum-based single-use plastics, for instance, gloves, protective medical suits, masks, hand sanitizer bottles, takeaway plastics, food containers, and medical test kits^1,2^. These conventional plastics are not biodegradable and may remain in landfills and marine for many years, significantly influencing soil quality, microbial activity, fauna, and flora^3^. As a result of entering the food chain, this causes a risk to human health^4^. Because of these concerns, biodegradable plastics with little or no environmental impact have gained popularity as alternatives to petroleum-based plastics. Besides, they are expected to be a part of future circular economies that will aid in achieving aspects of the UN's Sustainable Development Goals (SDGs)^5,6^.

Polyhydroxyalkanoates (PHAs) are polyesters produced in nature as an intracellular storage compound accumulated as energy reserve by some bacteria and archaea under stress conditions^7,8^. PHAs are thermoplastics with properties comparable to traditional petroleum-based polymers such as polypropylene (PP) and polyethylene (PE)^9,10^. Interestingly, the polymer in the PHAs family demonstrates outstanding biodegradability even when exposed to seawater^11^, making PHAs a promising replacement for petroleum-based plastics. PHAs are classified into two groups based on the carbon numbers in the monomeric constituents: short-chain-length PHAs (SCL-PHA, C3-C5), which consist of 3–5 carbon monomers, and medium-chain-length PHAs (MCL-PHA, C6-C14), which consist of 6–14 carbon monomers in the 3-hydroxyalkanoate units^12,13^.

Currently, more than 150 types of PHAs have been identified^14^, including homopolymers and copolymers, for instance, poly(3-hydroxybutyrate) [P(3HB], poly(3-hydroxybutyrate-*co*-4-hydroxybutyrate) [P(3HB-*co*-4HB)], poly(3-hydroxybutyrate-*co*-3-hydroxyvalerate) [P(3HB-*co*-3HV)], poly(3-hydroxybutyrate-*co*-3-hydroxyhexonate) [P(3HB-*co*-3HHx)]. Among PHA copolymers, Poly(3-hydroxybutyrate-*co*-3-hydroxy hexanoate) [P(3HB-*co*-3HHx)] is remarkably desirable due to its superior flexibility and resemblance to various popular petroleum-based polymers, which makes it more applicable to practical applications than stiff P(3HB) homopolymer^15,16^. Besides, due to its excellent biocompatibility and biodegradability, P(3HB-*co*-3HHx) is a suitable candidate copolymer for biomedical applications^17^.

Nevertheless, the commercial uses of polyhydroxyalkanoates (PHAs) have been constrained by the relatively high cost of manufacture compared to rival petrochemical polymers. Thus, the fermentation medium improvement is a critical field of research since its significant impact on both the growth of cells and the expression of desirable metabolites, contributing to total productivity^18^. Response surface methodology (RSM) is a statistical optimization approach that employs experimental factorial designs, such as central composite design (CCD) and Box-Behnken design (BBD), which are the major response surface designs to optimize process yield and specifies the response's behavior in the specified design space^19,20^. Both designs examine the interaction impact of elements that substantially influence product development. CCD and BBD experimental runs are used for RSM to establish the mathematical model that relates process parameters to outcomes^21^. However, BBD typically requires fewer design points than CCD, which may result in a regression model of poorer quality.

The recombinant PHA-producing strains with genes encoding PHA synthesis enzymes from various bacteria have been developed to generate short chain length-medium chain length (SCL-MCL) PHAs more effectively^22^. Our research group recently engineered a new P(3HB-*co*-3HHx)-producing bacterial strain, *C. necator* PHB^−^4/pBBR_CnPro-*phaC*_*Rp*_^23,24^. This strain produced 3.1 ± 0.3 g/L of P(3HB-*co*-3HHx) copolymer containing 2 mol% of 3HHx monomer composition when using 10 g/L of crude palm kernel oil (CPKO) as a sole carbon source^24^. However, an optimal culture medium and conditions for PHA production by this strain need to be improved to maximize the yield of P(3HB-*co*-3HHx). Thus, this study aims to optimize the fermentation condition for *C. necator* PHB^−^4/pBBR_CnPro-*phaC*_Rp_ using RSM to improve the P(3HB-*co*-3HHx) production. Besides, the fermentation was performed in a 10 L stirred-tank bioreactor to scale up the P(3HB-*co*-3HHx) production. Finally, the properties of this polymer were evaluated to confirm that this polymer is promising for various applications.

## Results

### Optimization of P(3HB-*co*-3HHx) production by RSM

The optimal medium composition and cultivation condition for P(3HB-*co*-3HHx) production and the interaction effects of each parameter were determined using a three-variable-five-level CCD design. The CCD variables included CPKO concentration, g/L(X1), sodium hexanoate concentration, g/L(X2) and culture time, h(X3). The experimental results of P(3HB-*co*-3HHx) production and predicted responses are demonstrated in Table 1. The results revealed that the highest P(3HB-*co*-3HHx) production (Run 6), 3.54 g/L, was achieved when the concentration of CPKO, sodium hexanoate, and cultivation time were 15 g/L, 1.0 g/L, and 54 h, respectively. While the lowest P(3HB-*co*-3HHx) production (Run 3) was 0.53 g/L when the concentrations of CPKO, sodium hexanoate, and cultivation time were 5 g/L, 3.0 g/L, and 42 h, respectively. The CCD experiment outputs from multiple regression analyses were fitted to a second-order polynomial model. The following model was employed to fit P(3HB-*co*-3HHx) production in terms of coded variables.

$$\begin{aligned} {\text{Y }} = &\,{3}.0{8} + 0.{\text{5791X1}}{-}0.{\text{7214X2}} + 0.{\text{2578X3}}{-}0.0{7}00{\text{X1X2}}{-}0.{4}0{\text{75X1X3}}{-} \, 0.{195}0{\text{X2X3}} \hfill \\&{-}0.{\text{3261X1}}^{{2}} {-}0.{\text{3615X2}}^{{2}} {-}0.{\text{1812X3}}^{{2}} \hfill \\ \end{aligned}$$

where Y is the P(3HB-*co*-3HHx) production and X1, X2, and X3 are coded values of CPKO, sodium hexanoate, and cultivation time, respectively.

**Table 1:** Experimental design and result of central composite design (CCD) of response surface methodology.

Run no	Level			PHA concentration (g/L)		PHA content (%DCW)	Dry cell weight (g/L)
	X1	X2	X3	Observed	Predicted		
1	− 1	− 1	− 1	1.33	1.42	41.4	3.21
2	1	− 1	− 1	3.52	3.53	63.2	5.57
3	− 1	1	− 1	0.53	0.51	28.2	1.88
4	1	1	− 1	2.15	2.34	52.9	5.01
5	− 1	− 1	1	3.27	3.27	72.5	4.51
6	1	− 1	1	3.54	3.62	70.2	5.04
7	− 1	1	1	1.4	1.45	33.1	4.23
8	1	1	1	1.68	1.65	48.7	3.45
9	− 1.68	0	0	1.14	1.18	55.9	2.04
10	1.68	0	0	3.25	3.13	59.5	5.46
11	0	− 1.68	0	3.27	3.14	59.0	5.54
12	0	1.68	0	0.92	0.84	37.9	2.43
13	0	0	− 1.68	2.26	2.13	69.5	3.25
14	0	0	1.68	2.95	3.00	50.0	5.9
15	0	0	0	3.14	3.08	62.5	5.02
16	0	0	0	3.12	3.08	59.5	5.24
17	0	0	0	2.80	3.08	60.5	5.12
18	0	0	0	3.11	3.08	60.2	5.17
19	0	0	0	3.13	3.08	60.0	5.22
20	0	0	0	3.14	3.08	60.9	5.16

The F test and ANOVA for the response surface quadratic model confirmed the equation's statistical significance. R^2^ = 0.9885 was the determination coefficient in this study’s regression equation **(**Table 2). As a result, this model can account for approximately 98.85% of the variability in the dependent variable; the remaining 1.15% was influenced by other factors. While the modified R^2^, which considers the sample size and number of terms^25^, was 0.9782. R^2^ values are constantly between 0 and 1. The higher the R^2^, the more influential the model and the better it predicts the response^21^. *P*-values are used to assess the significance of each coefficient, which contributes to understanding the pattern of mutual interactions among the variables^26^. The stronger the significance of the corresponding coefficient^27^, the smaller the *P* value. The F test and the corresponding *P*-values were estimated, as shown in Table 2. The model indicates that the constant linear (X1, X2, X3), quadratic (X1^2^, X2^2^, X3^2^), and interaction terms (X1X3 and X2X3) are significant (*P* < 0.05) (Table 2). However, due to the *P*-value for all variables (X1, X2 and X3) was smaller than 0.0001, it barely indicates which variables are the most significant for P(3HB-*co*-3HHx) production.

**Table 2:** Analysis of variance table.

Source	Sum of squares	df	Mean square	F-value	*P*-value
Model	17.58	9	1.95	95.77	< 0.0001*
X1-CPKO	4.58	1	4.58	224.51	< 0.0001*
X2-Sodium hexanoate	7.11	1	7.11	348.42	< 0.0001*
X3-Cultivation time	0.9075	1	0.9075	44.49	< 0.0001*
X1X2	0.0392	1	0.0392	1.92	0.1958
X1X3	1.33	1	1.33	65.12	< 0.0001*
X2X3	0.3042	1	0.3042	14.91	0.0032*
X1^2^	1.53	1	1.53	75.13	< 0.0001*
X2^2^	1.88	1	1.88	92.31	< 0.0001*
X3^2^	0.4730	1	0.4730	23.18	0.0007*
Residual	0.2040	10	0.0204		
Lack of Fit	0.1137	5	0.0227	1.26	0.4036
Pure Error	0.0903	5	0.0181		
Cor Total	17.79	19			

R^2^ = 0.9885, Adj-R^2^ = 0.9782 *Statistically significant at 95% probability level.

The negative polynomial coefficient in interaction terms in this model suggests that the interaction is oppositional. The lack of fit F-value of 1.26 (Table 2) indicates that the lack of fit is not statistically significant compared to the standard error. This high lack of fit F-value has a 40.36 percent probability of occurring due to noise.

**Figure 1:**
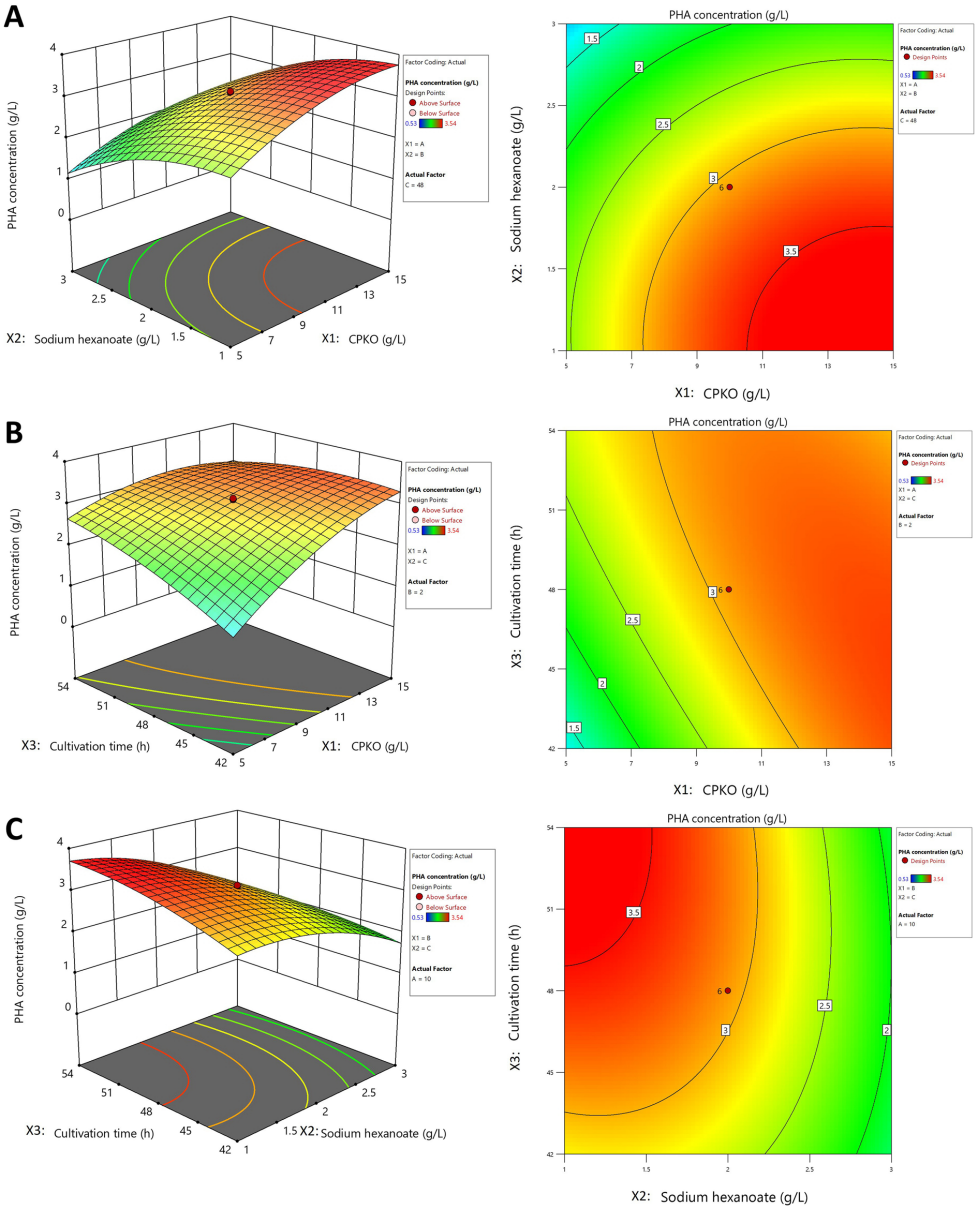
Response surface and contour plots described by the model, representing P(3HB-*co*-3HHx) production (g/L) as a value of CPKO, sodium hexanoate, and cultivation time by *C. necator* PHB^−^4/pBBR_CnPro-*phaC*_*Rp*_. The combined effect of CPKO and sodium hexanoate (**A**); CPKO and cultivation time (**B**); sodium hexanoate and cultivation time (**C**).

To evaluate the interaction between different parameters and to determine the optimal value of each parameter for maximum P(3HB-*co*-3HHx) production, the response between CPKO(X1), sodium hexanoate(X2) and cultivation time CPKO (X3) were plotted as shown in Fig. 1. Figure 1A shows the effect of CPKO and sodium hexanoate on P(3HB-*co*-3HHx) production. The P(3HB-*co*-3HHx) production increased when CPKO concentration increased from 5.0 to 15.0 g/L. At a lower CPKO concentration (< 5.0 g/L), P(3HB-*co*-3HHx) production declined. While P(3HB-*co*-3HHx) production increased with decreasing sodium hexanoate concentration, from 3.0 to 1.0 g/L. At a higher sodium hexanoate concentration (> 3.0 g/L) P(3HB-*co*-3HHx) production dramatically declined.

According to the RSM 3D graphs and 2D contour plots of CPKO(X1) and cultivation time(X2) on P(3HB-*co*-3HHx) production (Fig. 1B), it was shown that the P(3HB-*co*-3HHx) production significantly improved when CPKO was increased from 5.0 to 15.0 g/L. At the same time, it was reduced when the CPKO concentration declined to below 5.0 g/L. Besides, the production of P(3HB-*co*-3HHx) rose when the cultivation time was down from 54 to 42 h. Nevertheless, as cultivation time increased (> 54 h), P(3HB-*co*-3HHx) production dramatically decreased.

The effect of sodium hexanoate and cultivation time are shown in Fig. 1C. P(3HB-*co*-3HHx) production increased with decreased sodium hexanoate, from 3.0 to 1.0 g/L. While P(3HB-*co*-3HHx) production dramatically decreased at a higher concentration of sodium hexanoate (> 3.0 g/L) and increased with extended time, from 42 to 54 h. In addition, P(3HB-*co*-3HHx) production declined when cultivation time was lesser than 42 h.

The model was verified for the three factors in the design space to validate the optimization predictions. RSM-optimized medium composition and conditions were tested in triplicate on a 250-flask scale. The results demonstrate that under the following conditions: CPKO, 14.4 g/L, sodium hexanoate, 1.7 g/L, and 43 h of cultivation time, the maximum P(3HB-*co*-3HHx) production of 3.63 ± 0.4, with 5.54 ± 0.8 g/L of DCW was obtained, nearing the predicted P(3HB-*co*-3HHx) production of 3.55 g/L. The predicted and experimental values were compared, and the residual was calculated. The relative difference between the actual and predicted P(3HB-*co*-3HHx) production levels was 0.3%. As a result, the observed models are very accurate, and RSM analysis is an appropriate approach for predicting and improving fermentation medium and conditions.

### Scaling up of P(3HB-*co-*3HHx) production in a 10L bioreactor

**Figure 2:**
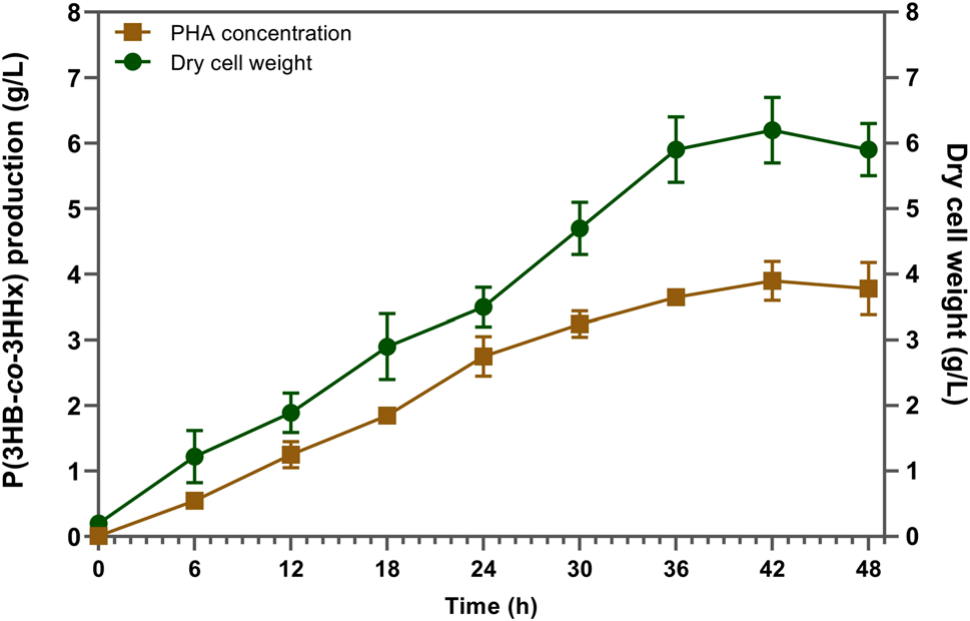
Batch fermentation profile of *C. necator* PHB^−^4/pBBR_CnPro-*phaC*_*Rp* p_ in 10L stirred-tank bioreactor.

Batch cultivation was carried out in a 10L stirred-tank bioreactor to enhance the cell biomass and P(3HB-*co*-3HHx) production of *C. necator* PHB^−^4/pBBR_CnPro-*phaC*_*Rp*_. The fermentation was carried out in a bioreactor containing 6 L of RSM-optimized media (CPKO, 14.4 g/L, sodium hexanoate, 1.7 g/L). The temperature, pH, aeration rate, and agitation speed were fixed at 30 °C, 6.8, 0.25 vvm, and 200 rpm, respectively. The growth and P(3HB-*co*-3HHx) production of *C. necator* PHB^−^4/pBBR_CnPro-*phaC*_*Rp*_ slowly increased during 48 h fermentation. As seen in Fig. 2, biomass increased gradually over fermentation. However, when the fermentation period was extended above the optimum (42 h), P(3HB-*co*-3HHx) production and cell growth were interrupted and the degradation of P(3HB-*co*-3HHx) began^28^. The highest production of P(3HB-*co*-3HHx) was at 42 h when the DCW was 6.2 ± 0.3 g/L; P(3HB-*co*-3HHx) production was 3.9 ± 0.3 g/L (Fig. 2). Besides, it should be noted that the higher 3HHx monomers fraction (5 mol%) was obtained when culturing *C. necator* PHB^−^4/pBBR_CnPro-*phaC*_*Rp*_ in a 10L stirred-tank bioreactor.

### P(3HB-*co-*3HHx) copolymer characterization

**Figure 3:**
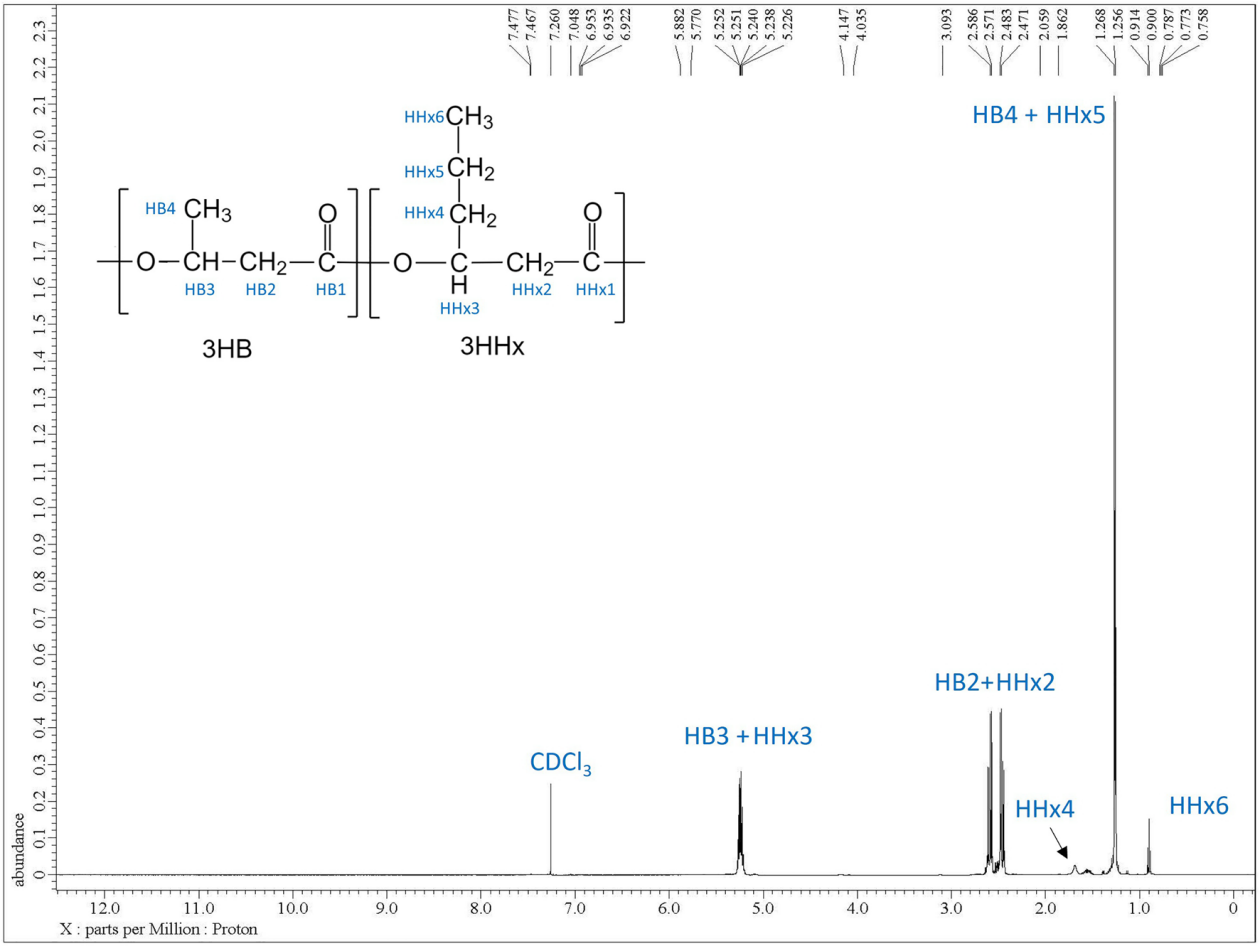
Proton Nuclear Magnetic Resonance Spectroscopy (^1^H NMR) spectrum of P(3HB-*co*-3HHx) produced by *C. necator* PHB^−^4/pBBR_CnPro-*phaC*_*Rp*_ in 10L stirred-tank bioreactor.

The extracted P(3HB-*co*-5 mol% 3HHx) copolymer produced from *C. necator* PHB^−^4/pBBR_CnPro-*phaC*_*Rp*_ was characterized by ^1^H NMR, FTIR, DSC and TGA to understand the copolymer's structural and thermal characteristics for further applications. The ^1^H NMR was performed to verify the presence of 3HHx monomer in the copolymer synthesized by the strain *C. necator* PHB^−^4/pBBR_CnPro-*phaC*_*Rp*_. Figure 3 illustrates the ^1^H NMR band of H4, corresponding to the C4 methylene groups, and the ^1^H NMR band of H6, corresponding to the C6 methyl group, indicating the formation of the P(3HB-*co*-3HHx) copolymer^15,24,29^. The monomer fractions of the copolymer were calculated according to the ^1^H spectrum intensity ratio of the methyl components^30^. The values of the 3HHx monomer fractions produced were slightly higher than those observed by gas GC analysis, with a variation of 1 mol%.

**Figure 4:**
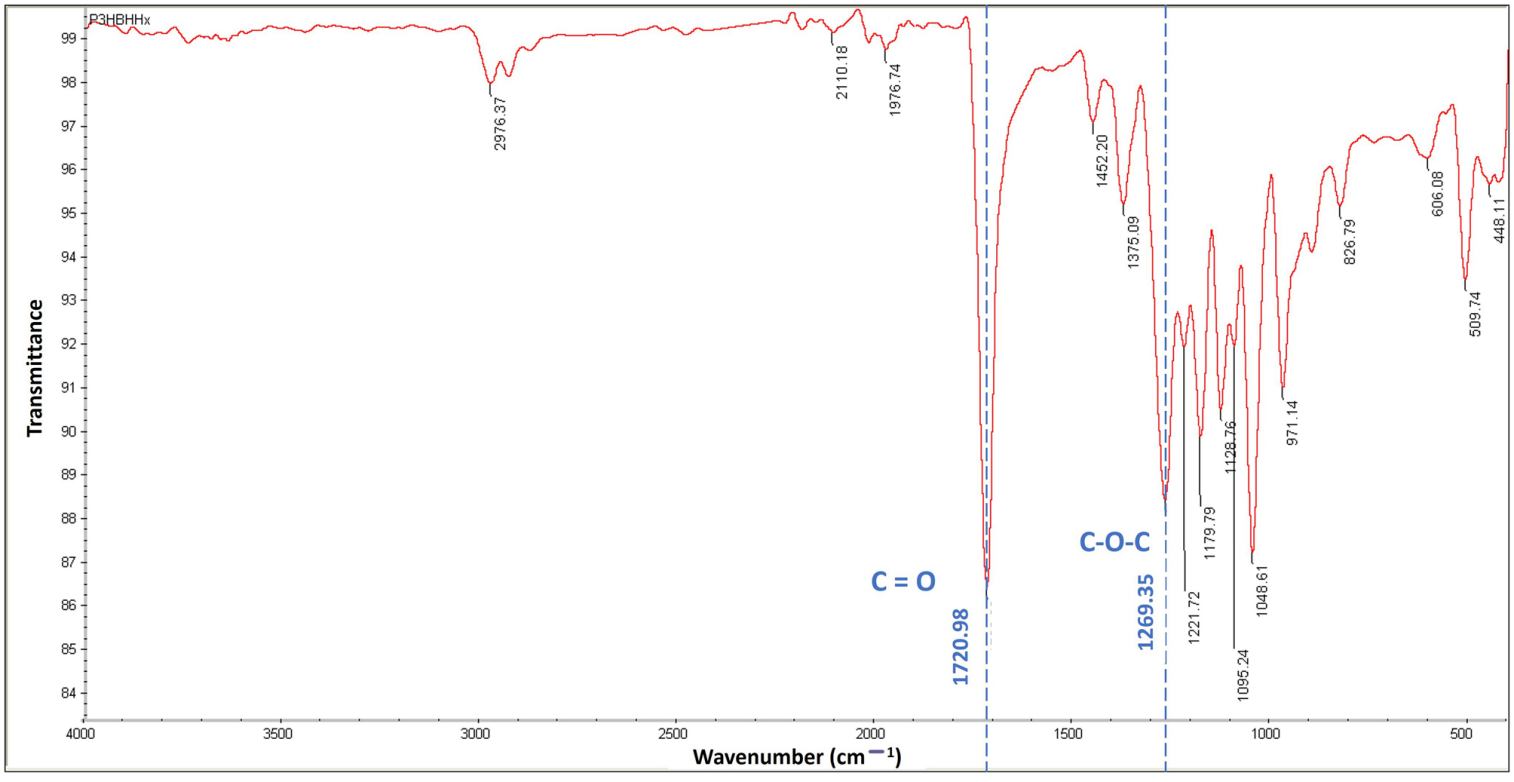
Attenuated Total Reflection Fourier transform infrared spectroscopy (ATR-FTIR) spectrum of P(3HB-*co*-3HHx) produced by *C. necator* PHB^−^4/pBBR_CnPro-*phaC*_*Rp*_ in 10L stirred-tank bioreactor.

FTIR absorption spectra were scanned in the range of 4000–400 cm^−1^. The FTIR spectra of copolymer synthesized by *C. necator* PHB^−^4/pBBR_CnPro-*phaC*_*Rp*_ are depicted in Fig. 4. The main absorption peak of P(3HB-*co*-3HHx) was observed in the spectrum at 1720.98 cm^−1^, which corresponds to the stretching vibration of the carbonyl (C=O) ester bond^31,32^. While the asymmetric C–O–C stretching vibration causes the absorption peak at 1269.35 cm − 1^33^. The C–H stretching and the –CH group were represented by the other distinctive bands located at 2976.37 cm^−1^ and 1221.72–1375.09 cm^−1^, respectively^34,35^. For the amorphous phase, the C–O and C–C stretching vibrations were attributed to a series of absorption bands ranging from 1179.79 to 606.08 cm^−1^^33^.

**Figure 5:**
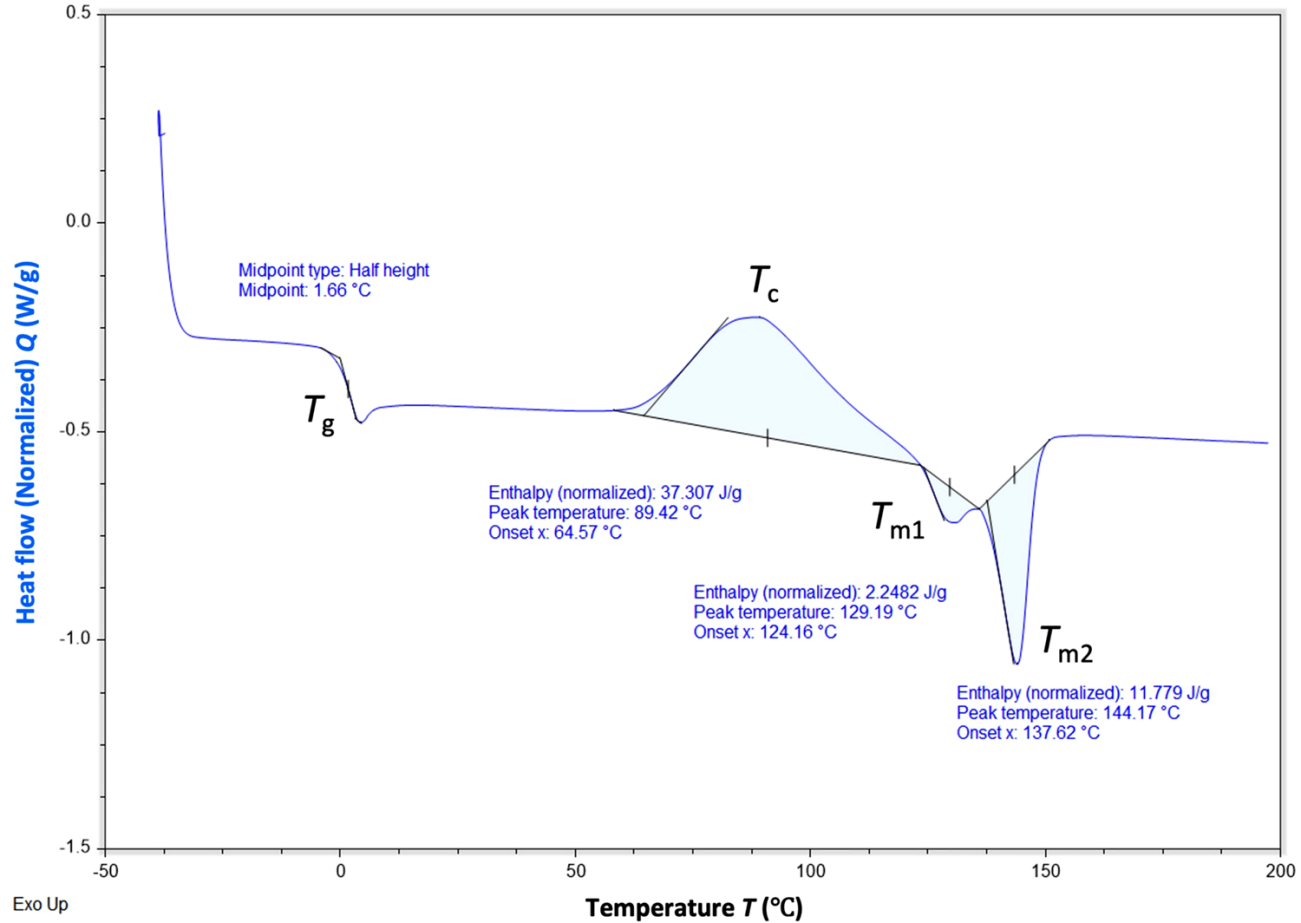
Differential Scanning Calorimetry (DSC) analysis of P(3HB-*co*-3HHx) produced by *C. necator* PHB^−^4/pBBR_CnPro-*phaC*_*Rp*_ in 10L stirred-tank bioreactor.

**Figure 6:**
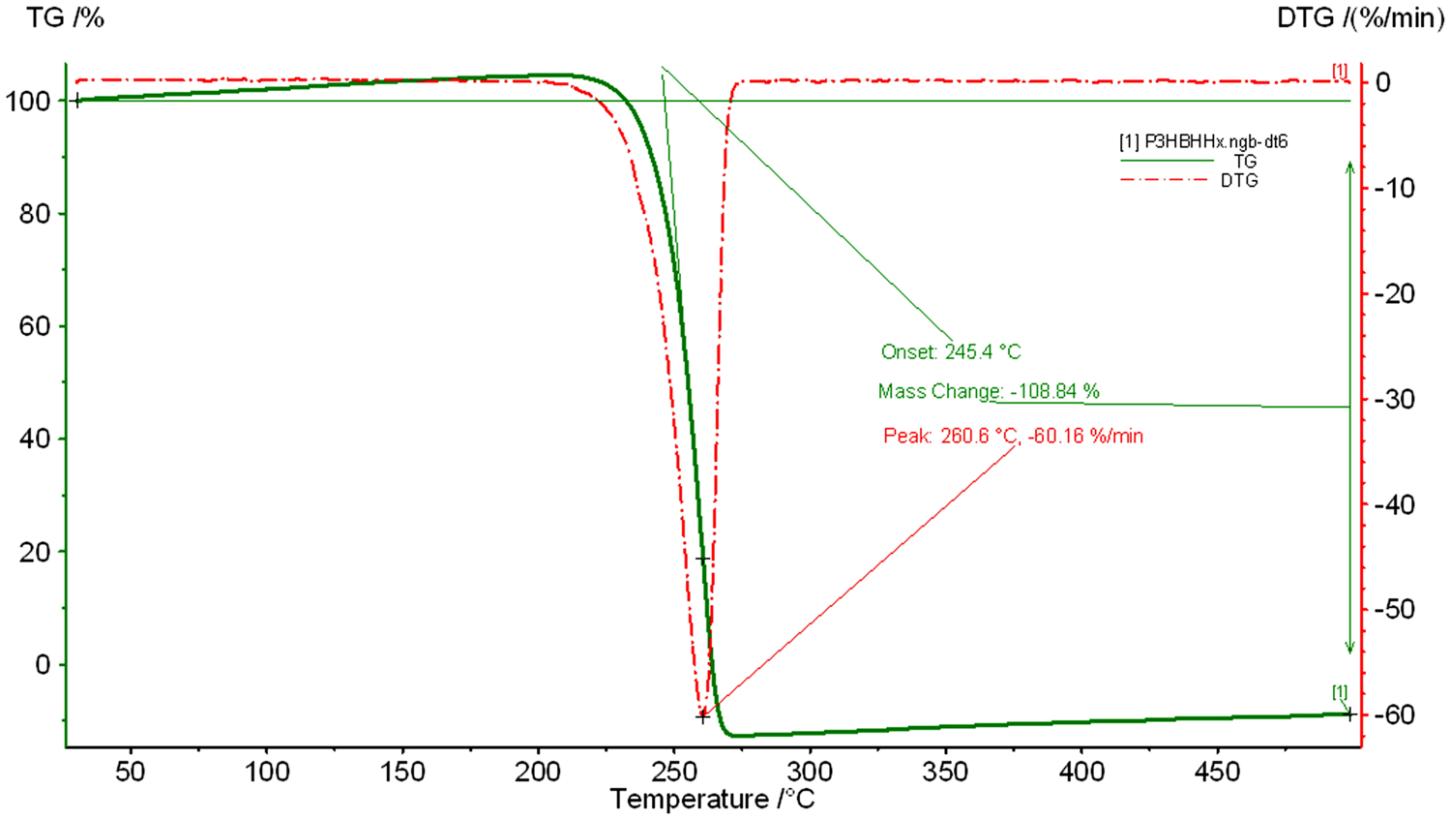
Thermogravimetric analysis (TGA) of P(3HB-*co*-3HHx) produced by *C. necator* PHB^−^4/pBBR_CnPro-*phaC*_*Rp*_ in 10L stirred-tank bioreactor.

Thermal properties of the P(3HB-*co*-5 mol% 3HHx) copolymer produced by *C. necator* PHB^−^4/pBBR_CnPro-*phaC*_*Rp*_ were analyzed using DSC and TGA. Figure 5 demonstrates the thermogram of melting temperature (*T*_m_), glass transition temperature (*T*_g_), while, and Fig. 6 shows the degradation temperatures (*T*_d_) of the copolymer. The values were recorded from the second heating to eliminate the thermal history of the previous samples. The thermogram of extracted copolymer revealed two melting temperatures (*T*_m1_ and. *T*_m2_) at approximately 129 and 144 °C (Fig. 5). The *T*_c_, *T*_g_ and *T*_d_ of the copolymer were around 89, 1.6 (Fig. 5) and 260.6 °C (Fig. 6), respectively.

## Discussion

PHAs are biodegradable polymeric materials produced from bacteria and archaea under stress conditions such as nutrient-limiting concentrations of nitrogen, phosphorus, sulfur, or oxygen and excess carbon sources^7,8,36^. Apparently, PHAs are becoming an increasingly significant issue in the scientific community as well as the business sector. This makes it possible for synthetic polymers to be replenished, ultimately establishing the intended circular economy. P(3HB-*co*-3HHx) is a practical type of PHA copolymer. It has a lower melting temperature and crystallinity than P(3HB) homopolymer, which can be attributed to the 3-hydroxyhexanate (3HHx) unit's long side chain^37^. The copolymers are composed of 5–15 mol% 3HHx and have elastic properties that make them suitable for various applications^38^.

Previously, Han et al. reported that the wild-type strains, *Aeromonas* spp., can produce P(3HB-*co*-3HHx) from vegetable oils and fatty acids via PHA synthase, which has a uniquely broad substrate specificity to (R)-3-hydroxyacyl-CoAs of C4-C6^39^. In this study, the P(3HB-*co*-3HHx) copolymer was synthesized by *C. necator* PHB^−^4/pBBR_CnPro-*phaC*_*Rp*_^23,24^, a newly engineered bacterial strain, and the P(3HB-*co*-3HHx) production was improved using RSM. The results showed that the highest 3.6 ± 0.4 g/L of P(3HB-*co*-4 mol% 3HHx) was obtained using the RSM-optimized conditions in the flask scale. Besides, the 3HHx monomer composition was increased to 5 mol% when cultivation *C. necator* PHB^−^4/pBBR_CnPro-*phaC*_*Rp*_ in the 10L bioreactor during 48 h of fermentation. This result was similar to the result achieved by Ouyang et al.^40^. They carried out the shake-flask experiments to produce P(3HB-*co*-3HHx) from *Aeromonas hydrophila,* which controllable monomer composition from 15% in the wild type to 3–12% in the recombinant by basically changing the ratio of gluconate to lauric acid in the culture media in 48 h of fermentation. The P(3HB-*co*-3HHx) production studied in *Cupriavidus* sp*.* has been reported by Volova et al.^22^, *C. eutrophus* B10646 could produce significant biomass yields (5.6 g L^−1^), and a high content of the polymer (60–75%), including a high 3HHx molar fraction, under the proper growth conditions. However, the physicochemical and mechanical characteristics of P(3HB-*co*-3HHx) copolymers can be changed by adjusting the 3HB/3HHx ratio. Besides, Kawashima et al.^41^ found that the region downstream of phaP1Re was an advantageous site for integrating genes that are overexpressed during PHA accumulation in *R. eutropha*. The findings also demonstrated that the polymerization characteristics of PHA synthase were influenced by the kind of phasin that coexisted on the surface of PHA granules, altering the resultant PHA polymer (3HB-*co*-3HHx). The replacement of phasin is an innovative technical method for controlling the composition of PHA copolyesters. Furthermore, Murugan et al.^42^ investigated palm olein (PO) and fructose as carbon sources for the biosynthesis of P(3HB-*co*-3HHx) by recombinant *C. necator* Re2058/pCB113. Shake flask cultures utilizing 5 g/L PO as the only carbon source yielded a cell dry weight (CDW) of 5.13 g/L, 67% PHA/CDW, and a copolymer comprising 27 mol% 3HHx. P(3HB-*co*-3HHx) with 4–15 mol% 3HHx monomer had molecular weights in the range of 5.47–6.85 × 10^5^ Da, which was at least two-fold more than previously reported values.

In this study, RSM employing CCD was applied to improve the production of P(3HB-*co*-3HHx) in a flask scale. The result shows that under the following conditions: CPKO, 14.4 g/L, sodium hexanoate, 1.7 g/L, and 43 h of cultivation time, the maximum P(3HB-*co*-3HHx) production of 3.63 ± 0.4 was obtained, nearing the RSM predicted P(3HB-*co*-3HHx) production of 3.55 g/L. These results proved the accuracy of the RSM model for P(3HB-*co*-3HHx) production. Furthermore, compared to the unoptimized condition, the optimized medium can improve the production of P(3HB-*co*-3HHx) and 3HHx monomer composition by 1.2 and twofold, respectively. Based on this, the improvement of P(3HB-*co*-3HHx) production was slightly present, however, the 3HHx monomer composition was successfully enhanced by RSM. Previously, RSM was reported to be an effective method for improving PHA biosynthesis by many microorganisms^43–47^. However, the use of RSM to improve P(3HB-*co*-3HHx) production has been limited.

The application of batch fermentation by various bacteria to increase PHA biosynthesis in the bioreactor has been reported^48–52^. However, in this present study, P(3HB-*co*-3HHx) production cannot be enhanced using this approach. Only the 3HHx monomer's composition was increased to 5 mol%, which may be related to the reduced harvesting period. In addition, batch cultivations are easy to operate yet have poor intrinsic productivity due to the restricting concentration of carbon and nitrogen supplies at the beginning of fermentation^53^. In contrast, the fed-batch fermentation method produces a high cell concentration, improves productivity, and reduces substrate or end-product inhibition^54^.

In this study, the P(3HB-*co*-3HHx) copolymer containing 5 mol% 3HHx monomer composition was synthesized by the engineered strain, *C. necator* PHB^−^4/pBBR_CnPro-*phaC*_*Rp*_ using CPKO and sodium hexanoate as a carbon and precursor, respectively. The copolymer was extracted and characterized to understand the structural and thermal characteristics of the copolymer for further use. The ^1^H NMR spectrum confirmed the existence of 3HHx monomer in P(3HB-*co*-3HHx) copolymers synthesized by *C. necator* PHB^−^4/pBBR_CnPro-*phaC*_*Rp*_. It was similar to the ^1^H NMR spectra reported by Wong et al.^30^ and Bhubalan et al.^55^. Besides, according to the 3HHx monomer composition calculation from ^1^H NMR spectra, this polymer contains 5–6 mol% 3HHx monomer, similar to the GC analysis results. The FTIR spectra of copolymer demonstrated the characteristic absorption peak of P(3HB-*co*-3HHx) copolymers at 1720.98 and 1269.35 cm^−1^, which corresponds to the stretching vibration of the carbonyl (C=O) ester bond and asymmetric C–O–C stretching vibration, respectively^31,32^. These results established that the produced copolymer was P(3HB-*co*-3HHx).

Thermal properties of the P(3HB-*co*-5 mol% 3HHx) copolymer produced by *C. necator* PHB^−^4/pBBR_CnPro-*phaC*_*Rp*_ were analyzed using DSC and TGA. The DSC thermogram of extracted copolymer revealed two melting temperatures (*T*_m1_ and. *T*_m2_) at approximately 129 and 144 °C. Two melting temperatures for mcl-PHAs are detectable, which may be related back to the formation of two different crystal phases (phase I and phase II)^56^. The *T*_c_, *T*_g_ and *T*_d_ of the copolymer were 89, 1.6 and 260.6 °C, respectively. These results are similar to the previously studied by Murugan et al.^42^. They have reported that the *T*_m_ and *T*_g_ of P(3HB-*co*-4 mol% 3HHx) produced from *C. necator* Re2058/pCB113 were 164 and − 1 °C, respectively.

The P(3HB)* T*_d_ was reported at 280 °C^56–58^. The *T*_d_ copolymer in this study was lower due to the incorporation of the 3HHx monomer. In general, P(3HB-*co*-3HHx) copolymers had a lower *T*_m_ and *T*_d_ than P(3HB), although there was no discernible association between these characteristics and the 3HHx molar fractions^22^.

## Conclusions

In this study, the production of the P(3HB-*co*-3HHx) copolymers by an engineered strain of *C. necator* PHB^−^4/pBBR_CnPro-*phaC*_*Rp*_ was improved using RSM. Under RSM optimum conditions, this strain can produce 3.6 ± 0.4 of P(3HB-*co*-3HHx) containing 4 mol% 3HHx compositions. Compared to the unoptimized condition, the optimized medium can improve the production of P(3HB-co-3HHx) and 3HHx monomer composition by 1.2 and twofold, respectively. Besides, Interestingly, the 3HHx monomer composition was enhanced to 5 mol% when operating the fermentation in a 10 L stirred-tank bioreactor, which was 2.5-fold higher than the unoptimized condition. The functional group and chemical structure results verified the polymer as P(3HB-*co*-3HHx), and the produced polymer's thermal properties were similar to industrial P(3HB-*co*-3HHx).

## Materials and methods

### Bacterial strain and inoculum preparation

The recombinant strain *C. necator* PHB^−^4/pBBR_CnPro-*phaC*_*Rp*_ was cultivated as described by Trakunjae et al.^24^. Briefly, the bacterial strain was cultured on nutrient-rich (NR) agar supplemented with 50 µg/mL kanamycin at 30 °C for 24 h. Then, three full loops of a bacterial colony were transferred in NR medium supplemented with 50 µg/mL kanamycin to prepare the bacterial inoculum. After that, incubated the inoculum flasks at 30 °C with shaking of 200 rpm for 8 h or until the optical density (OD600) reached 4.

### Biosynthesis of P(3HB-*co*-3HHx) copolymers

The 3% v/v of *C. necator* PHB^−^4/pBBR_CnPro-*phaC*_*Rp*_ inoculum was transferred into the P(3HB-*co*-3HHx) production medium. The mineral medium (MM) for P(3HB-*co*-3HHx) production consisted of 0.45 g/L of K_2_SO_4_, 4.6 g/L of Na_2_HPO_4_, 4.0 g/L of NaH_2_PO_4_, 0.54 g/L of CO(NH_2_)_2_ [Urea], 0.39 g/L of MgSO_4_, 0.062 g/L of CaCl_2_ and 1 mL/L of trace element (TE) solution^59^. The TE solution comprised of ZnSO_4_·7H_2_O, 2.4 g/L; FeSO_4_·7H_2_O, 15 g/L; MnSO_4_·H_2_O, 2.4 g/L, and CuSO_4_·5H_2_O, 0.48 g/L dissolved in 0.1 M HCl. The pH of MM was adjusted to 6.8 prior to sterilization. CPKO, sodium hexanoate, and CaCl_2_ were sterilized separately at 121 °C for 20 min. While urea and TE solution were filtered using a 0.2 um sterile membrane filter and added to the sterilized medium at the required concentration. The P(3HB-*co*-3HHx) biosynthesis of *C. necator* PHB^−^4/pBBR_CnPro-*phaC*_*Rp*_ was carried out at 30 °C with shaking of 200 rpm for 48 h.

### Harvesting of bacterial cells

The bacterial cells were harvested by centrifugation at 8,000 rpm, 4 °C for 10 min. After that, the cell pellets were washed with distilled water (DW), followed by a mixed solution of DW and hexane in a ratio of 1:1 to remove the oil residues. Next, the cell pellets were rewashed with DW to remove the hexane remains and transferred to a pre-weighed bijoux bottle. Then, the bottles containing bacterial cell pellets were frozen at − 20 °C overnight and lyophilized using a freeze-dryer until completely dry. Finally, the weight of the lyophilized cell was recorded in g/L. At the same time, the PHA content and monomer composition were examined by gas chromatography (GC) analysis.

### Optimization of P(3HB-*co*-3HHx) copolymers using RSM

RSM, a practical modeling method, is a set of statistical and mathematical tools for creating experiments and optimizing the influence process variables^60^. In this study, the production of P(3HB-*co*-3HHx) copolymers was enhanced using the RSM based on central composite design (CCD). It is commonly used to construct a second-order polynomial for the response variables without a full factorial design of experiments.

Three significant factors were used in this study, i.e., CPKO (g/L) (X1), sodium hexanoate (g/L) (X2), and cultivation time (h) (X3). Each variable was coded at five levels (1.68, 1, 0, + 1, and + 1.68) based on the CCD design to define the characteristics of the response surface in the optimal region. A total of twenty fermentation runs were designed according to Eq. (1), including five replicated fermentation runs at the center points.

1 $${\text{Total number of experiments}} = {\text{ k}}^{{2}} \, + \,{\text{2k}}\, + \,{\text{n}}0$$

where k is the number of independent variables and n0 is the number of repetitions of experiments at the center point

The coded and actual levels of the significant factors are presented in Table 3. The design matrix of the tested fermentation runs is demonstrated in Table 1. The average values were reported from triplicate experimental runs. Design-Expert v7.0.0 software (Stat-Ease, Inc. MN, USA) was used for the statistical analysis of the results. The experimental results of the CCD design were fitted with a second-order polynomial equation by multiple regression techniques, as shown in Eq. (2).

2 $$Y = \beta_{0} + \sum\limits_{i = 1}^{k} {\beta_{i} X_{i} + } \sum\limits_{i < } {\sum\limits_{j = 2}^{k} {\beta_{li} X_{i} X_{j} } }$$

where *Y* is the predictive measured response; *X*_i_ and *X*_j_ are the independent variables; *β*_0_ represents the intercept; and *β*_i_, *β*_ii_, and *β*_lj_ are the regression coefficients of the model^61^. The generated model for three independent variables is shown in Eq. (3).

3 $$Y = \beta_{0} + \beta_{1} X_{1} + \beta_{2} X_{2} + \beta_{3} \beta_{3} + \beta_{11} X_{1}^{2} + \beta_{22} X_{2}^{2} + \beta_{33} X_{3}^{2} + \beta_{12} X_{1} X_{2} + \beta_{13} X_{1} X_{3} + \beta_{23} X_{2} X_{3}$$

where *Y* is the predicted response of P(3HB-*co*-3HHx) production (g/L); *β*1, *β*2, and *β*3 are linear coefficients; *β*11, *β*22, and *β*33 represent quadratic coefficients; *β*12, *β*13, and *β*23 are interaction coefficients; X1, X2, and X3 represent coded values of CPKO (X1), sodium hexanoate (X2) and cultivation time (X3).

**Table 3:** Experimental code and actual levels.

Independent variables	Unit	Range and levels				
		− 1.68	− 1.00	0.00	+ 1.00	+ 1.68
CPKO, X1	g/L	1.59	5	10.0	15	18.4
Sodium hexanoate, X2	g/L	0.31	1	2.0	3	3.68
Cultivation time, X3	h	37.9	42	48	54	58.1

### Validation of the RSM model

The values of the three tested factors, CPKO, sodium hexanoate, and cultivation time, were chosen randomly from the design space to verify the P(3HB-*co*-3HHx) production by *C. necator* PHB^−^4/pBBR_CnPro-*phaC*_*Rp*_ in a shake flask model. In this experiment, the other components of the medium were at fixed levels.

### Scaling up of P(3HB-*co-*3HHx) production in a 10L bioreactor

Fermentation was carried out in a 10 L stirred-tank bioreactor (Model MDFT-N-10L, Marubishi, Japan) to improve the production of P(3HB-*co*-3HHx) by *C. necator* PHB^−^4/pBBR_CnPro-*phaC*_*Rp*_. The 3% v/v of bacterial inoculum was transferred to the bioreactor containing 6 L of optimized media. Batch cultivation was carried out at 30 °C with initial pH and agitation speeds of 6.8 and 200 rpm, respectively. The pH of the culture broth was maintained at pH 6.8 during the fermentation by adding HPO_3_ or NaOH using a pH controller. The airflow rate was fixed at 0.25 vvm. The cell biomass and P(3HB-*co*-3HHx) production were evaluated every 6 h during 48 h of fermentation. The fermentations were performed in triplicates and average values were determined.

### P(3HB-*co*-3HHx) copolymers extraction and purification

The 10 g of freeze-dried cells were dissolved in 1 L chloroform and stirred for 3–5 days at room temperature to extract the P(3HB-*co*-3HHx) copolymers. Then, the cell debris was removed by filtering the bacterial cell suspension using filter paper (Whatman No. 1). After that, the P(3HB-*co*-3HHx) dissolved chloroform solution was evaporated to approximately 100 mL using a rotary evaporator. The evaporated solution was subsequently added drop by drop to 100 mL of ice-cold methanol and stirred for 1 h. Finally, the purified polymer was separated by filtration using 0.45 µm PTFE membrane and air dried for 3–5 days^44^ before being used for further experiments.

### Characterization of P(3HB-*co*-3HHx) copolymers

Proton nuclear magnetic resonance (^1^H NMR) spectroscopy is a simple technique to investigate PHA polymer composition. In this study, the purified P(3HB-*co*-3HHx) copolymers were dissolved in deuterated chloroform (CDCl_3_) at 25 mg/mL to apply for NMR analysis. The solution-state ^1^H NMR was carried out on a Jeol JNM-ECZ-400R/S1 spectrophotometer (JEOL, Ltd., Tokyo, Japan) resonating at 500 MHz. The chemical shifts were referred to the tetramethylsilane (TMS). At the same time, adamantane was used as an external standard.

The functional groups of purified P(3HB-*co*-3HHx) copolymers were detected by Fourier transform IR (FTIR) spectroscopy. The FTIR analysis was performed using an FTIR spectrometer (Thermo Scientific Nicolet IR200, Waltham, MA, USA). The 128 scans were composed in attenuated total reflection (ATR) mode. Besides, the spectra were achieved in the range of 4000 to 400 cm^−1^ with a resolution of 4 cm^−1^.

The purified P(3HB-*co-*3HHx) copolymers were analyzed for their thermal properties using differential scanning calorimetry (DSC) and thermogravimetric analysis (TGA). DSC analysis was analyzed by DSC25 (TA instruments, New Castle, DE, USA) using a nitrogen flow rate of 30 mL/min. Around 3–5 mg of purified P(3HB-*co*-3HHx) copolymers were filled into a Tzero Aluminum Hermetic pan, covered, and heated from 25 to 200 °C at a heating rate of 15 °C/min. The melted samples were then maintained at 200 °C for 2 min and rapid reduction to − 40 °C. Finally, they were repeatedly heated from − 40 to 200 °C at a heating rate of 15 °C/min. The melting temperature (*T*_m_), crystallization temperature (*T*_c_), and glass transition temperature (*T*_g_) were detected and analyzed from the DSC thermogram. For TGA analysis, approximately 5 mg of the purified P(3HB-co-3HHx) copolymers were filled in an aluminum pan and analyzed using Pyris 1 TGA instrument (Perkin Elmer, USA). The heating temperature was set from 30 to 900 °C at a heating rate of 20 °C/min under a nitrogen atmosphere.

### Analysis of dry cell weight (DCW)

The determination of DCW was modified from Trakunjae et al.^24^. Briefly, 1 mL of cell culture suspension was transferred into the pre-weighed Eppendorf tubes and centrifuged at 8,000 rpm for 10 min. Then, the harvested cells were washed with distilled water, followed by a mixed solution of DW and hexane in a ratio of 1:1 to remove the oil residues. Then washed with DW to remove hexane remains and centrifuged at 8,000 rpm for 10 min. Next, the obtained cell pellets were frozen at − 20 °C overnight and lyophilized using a freeze-dryer for 2–3 days. Finally, the Eppendorf tubes containing lyophilized cells were weighed to verify stability and calculated the DCW in g/L.

### Analysis of PHA content

The PHA content and monomers composition were analyzed using the methanolysis technique following Braunegg et al.^62^. Briefly, 15–20 mg of lyophilized cells were added to the test tube, followed by 2 mL of chloroform and methanolysis solution (mixture of 85% v/v of methanol and 15% w/v H_2_SO_4_). The tubes were heated at 100 ° C for 180 min, then cooled at room temperature. After that, 1 mL of DW was added into the tubes and mixed vigorously for 1 min using a vortex mixer. The chloroform-rich PHA in the bottom layer was collected using a pasture pipette. Then, remove the water residues using Na_2_SO_4_. The mixture solution of 500 mL of chloroform-rich PHA solution and 500 mL of 0.2% (v/v) caprylic methyl ester (CME) (Internal standard) was prepared for GC analysis. The analysis was performed using Shimadzu GC-2014 plus (Shimadzu, Japan) supplied with Restek RTX-1 column (Restek, USA) and flame ionization detector (FID). The 2.0 µL of the prepared sample solution was injected into the GC machine. Nitrogen was used as a carrier gas for GC analysis. Besides, the injector and detector temperatures were set at 270 °C and 280 °C, respectively.

### Statistical analysis

All experimental data were described as mean ± standard error. The statistical analysis was carried out by SPSS statistics 17.0 software (SPSS for Windows, SPSS Inc., Chicago, IL, USA). Experimental responses were examined using a two-way analysis of variance (ANOVA). Each model term's linear, quadratic, and interaction regression coefficients were calculated using the F-value at a probability (*P*) < 0.05. In addition, the statistical significance of each term in the polynomial was analyzed, and all coefficients were investigated using Design-Expert® v7.0.0 software (Stat-Ease, Inc. MN, USA).

## Data Availability

All data generated or analysed during this study are included in this published article. Correspondence and requests for materials should be addressed to C. Trakunjae or P. Vaithanomsat.

## References

[1] Benson NU, Bassey DE, Palanisami T (2022). COVID pollution: Impact of COVID-19 pandemic on global plastic waste footprint. Heliyon..

[2] Wang Q, Zhang M, Li R (2022). The COVID-19 pandemic reshapes the plastic pollution research—A comparative analysis of plastic pollution research before and during the pandemic. Environ. Res..

[3] Nanda S (2022). Innovations in applications and prospects of bioplastics and biopolymers: A review. Environ. Chem. Lett..

[4] Smith M, Love DC, Rochman CM, Neff RA (2018). Microplastics in seafood and the implications for human health. Curr. Environ. Health Rep..

[5] Chen X, Kroell N, Li K, Feil A, Pretz T (2021). Influences of bioplastic polylactic acid on near-infrared-based sorting of conventional plastic. Waste Manag. Res..

[6] Karan H, Funk C, Grabert M, Oey M, Hankamer B (2019). Green bioplastics as part of a circular bioeconomy. Trends Plant Sci..

[7] Doi Y (1990). Microbial Polyesters.

[8] Byrom, D. Polyhydroxyalkanoates. In *Plastic from microbes: microbial synthesis of polymers and polymer precursors* (ed. Mobley, D. P.) 5–33 (Hanser, Munich, 1994).

[9] Lee SY (1996). Plastic bacteria? Progress and prospects for polyhydroxyalkanoate production in bacteria. Trends Biotechnol..

[10] Sudesh K (2013). Polyhydroxyalkanoates from Palm Oil: Biodegradable Plastic.

[11] Sashiwa H, Fukuda R, Okura T, Sato S, Nakayama A (2018). Microbial degradation behavior in seawater of polyester blends containing Poly(3-hydroxybutyrate-*co*-3-hydroxyhexanoate) (PHBHHx). Mar. Drugs.

[12] Li Z, Loh XJ (2015). Water soluble polyhydroxyalkanoates: Future materials for therapeutic applications. Chem. Soc. Rev..

[13] Basnett, P., Ravi, S. & Roy, I. 8 - Natural bacterial biodegradable medical polymers: Polyhydroxyalkanoates in *Science and Principles of Biodegradable and Bioresorbable Medical Polymers*, (ed. Zhang, X.) 257–277 (Woodhead Publishing, 2017).

[14] Kim DY, Kim HW, Chung MG, Rhee YH (2007). Biosynthesis, modification, and biodegradation of bacterial medium-chain-length polyhydroxyalkanoates. J. Microbiol..

[15] Tanaka K, Yoshida K, Orita I, Fukui T (2021). Biosynthesis of Poly(3-hydroxybutyrate-co-3-hydroxyhexanoate) from CO_2_ by a Recombinant *Cupriavidus necator*. Bioengineering.

[16] Doi Y, Kitamura S, Abe H (1995). Microbial synthesis and characterization of poly(3-hydroxybutyrate-*co*-3-hydroxyhexanoate). Macromolecules.

[17] Wu Q, Wang Y, Chen GQ (2009). Medical application of microbial biopolyesters polyhydroxyalkanoates. Artif. Cells Blood Substit. Biotechnol..

[18] Srivastava A (2018). Response surface methodology-genetic algorithm based medium optimization, purification, and characterization of cholesterol oxidase from *Streptomyces rimosus*. Sci. Rep..

[19] El-Naggar NEA, El-Shweihy NM, El-Ewasy SM (2016). Identification and statistical optimization of fermentation conditions for a newly isolated extracellular cholesterol oxidase-producing *Streptomyces cavourensis* strain NEAE-42. BMC Microbiol..

[20] Singh V, Tripathi CKM (2008). Production and statistical optimization of a novel olivanic acid by *Streptomyces olivaceus* MTCC 6820. Process Biochem..

[21] Aghaie E (2009). Response surface methodology (RSM) analysis of organic acid production for Kaolin beneficiation by *Aspergillus niger*. Chem. Eng. J..

[22] Volova TG, Syrvacheva DA, Zhila NO, Sukovatiya AG (2016). Synthesis of P(3HB-*co*-3HHx) copolymers containing high molar fraction of 3-hydroxyhexanoatemonomer by *Cupriavidus eutrophus* B10646. J. Chem. Technol. Biotechnol..

[23] Trakunjae C (2021). Enhanced polyhydroxybutyrate (PHB) production by newly isolated rare actinomycetes *Rhodococcus* sp. strain BSRT1–1 using response surface methodology. Sci Rep..

[24] Trakunjae C (2022). Biosynthesis of P(3HB-*co*-3HHx) copolymers by a newly engineered strain of *Cupriavidus necator* PHB^−^4/pBBR_CnPro-*phaC*_*Rp*_ for skin tissue engineering application. Polymers.

[25] Zhang YJ (2012). Optimization of succinic acid fermentation with *Actinobacillus succinogenes* by response surface methodology (RSM). J. Zhejiang Univ. Sci. B..

[26] Ram Kumar PS (2009). Optimization and fed-batch production of PHB utilizing dairy waste and sea water as nutrient sources by *Bacillus megaterium* SRKP-3. Bioresour. Technol..

[27] Qi BK (2009). Optimization of enzymatic hydrolysis of wheat straw pretreated by alkaline peroxide using response surface methodology. Ind. Eng. Chem. Res..

[28] Raza Z, Tariq M, Majeed M, Banat I (2019). Recent developments in bioreactor scale production of bacterial polyhydroxyalkanoates. Bioprocess Biosyst. Eng..

[29] Pieper U, Steinbüchel A (1992). Identification, cloning and sequence analysis of the poly(3-hydroxyalkanoic acid) synthase gene of the gram-positive bacterium *Rhodococcus ruber*. FEMS Microbiol. Lett..

[30] Wong YM, Brigham CJ, Rha C, Sinskey AJ, Sudesh K (2012). Biosynthesis and characterization of polyhydroxyalkanoate containing high 3-hydroxyhexanoate monomer fraction from crude palm kernel oil by recombinant *Cupriavidus necator*. Bioresour. Technol..

[31] Randriamahefa S, Renard E, Guérin P, Langlois V (2003). Fourier transform infrared spectroscopy for screening and quantifying production of PHAs by *Pseudomonas* grown on sodium octanoate. Biomacromol.

[32] Salim YS (2016). Evidence of melt reaction between poly(3-hydroxybutyrate-*co*-3-hydroxyhexanoate) and epoxidized natural rubber as investigated by DSC, isothermal TGA and FTIR analyses. Macromol. Symp..

[33] Gumel AM, Annuar MSM, Heidelberg T (2012). Biosynthesis and characterization of polyhydroxyalkanoates copolymers produced by *Pseudomonas putida* Bet001 isolated from palm oil mill effluent. PLoS ONE.

[34] Sathiyanarayan G (2017). Production and characterization of medium-chain-length polyhydroxyalkanoate copolymer from Arctic psychrotrophic bacterium *Pseudomonas* sp. PAMC 28620. Int. J. Biol. Macromol..

[35] Lopez-Cuellar MR, Alba-Flores J, Gracida-Rodríguez JN, Erez-Guevara FP (2011). Production of polyhydroxyalkanoates (PHAs) with canola oil as carbon source. Int. J. Biol. Macromol..

[36] Chen GQ (2009). A microbial polyhydroxyalkanoates (PHA) based bio- and materials industry. Chem. Soc. Rev..

[37] Li Z, Yang J, Loh X (2016). Polyhydroxyalkanoates: Opening doors for a sustainable future. NPG Asia Mater..

[38] Taguchi, S., Iwata, T., Abe, H. & Doi, Y. “Poly(hydroxyalkanoate)s,”. In *Polymer Science*: *A Comprehensive Reference*. (ed. Matyjaszewski, K. & Möller, M.), 157–182 (Elsevier, Amsterdam, 2012).

[39] Han J, Qiu Y-Z, Liu D-C, Chen GQ (2004). Engineered *Aeromonas Hydrophila* for enhanced production of Poly(3-Hydroxybutyrate-*co*-3-Hydroxyhexanoate) with alterable monomers composition. FEMS Microbiol. Lett..

[40] Ouyang SP, Qiu YZ, Wu Q, Chen GQ (2003). Fermentative production of poly(3-hydroxybutyrate-*co*-3-hydroxyhexanoate) (PHBHHx) by recombinant *Aeromonas hydrophila* 4AK4 (pTG01). Sheng Wu Gong Cheng Xue Bao. Chinese.

[41] Kawashima Y, Orita I, Nakamura S, Fukui T (2015). Compositional regulation of poly(3-hydroxybutyrate-*co*-3-hydroxyhexanoate) by replacement of granule-associated protein in *Ralstonia eutropha*. Microb. Cell Fact..

[42] Murugan P, Gan CY, Sudesh K (2017). Biosynthesis of P(3HB-*co*-3HHx) with improved molecular weights from a mixture of palm olein and fructose by *Cupriavidus necator* Re2058/pCB113. Int. J. Biol. Macromol..

[43] Campos MI, Figueiredo TVB, Sousa LS, Druzian JI (2014). The influence of crude glycerin and nitrogen concentrations on the production of PHA by *Cupriavidus necator* using a response surface methodology and its characterizations. Ind. Crops Prod..

[44] Ojha N, Das NA (2018). Statistical approach to optimize the production of Polyhydroxyalkanoates from *Wickerhamomyces anomalus* VIT-NN01 using response surface methodology. Int. J. Biol. Macromol..

[45] Hassan MA, Bakhiet EK, Hussein HR, Ali SG (2019). Statistical optimization studies for polyhydroxybutyrate (PHB) production by novel *Bacillus subtilis* using agricultural and industrial wastes. Int. J. Environ. Sci. Technol..

[46] Ronďošová S, Legerská B, Chmelová D, Ondrejovič M, Miertuš S (2022). Optimization of growth conditions to enhance PHA production by *Cupriavidus necator*. Fermentation.

[47] Narayanan A, Ramana K (2012). Polyhydroxybutyrate production in *Bacillus mycoides* DFC1 using response surface optimization for physico-chemical process parameters. 3 Biotech.

[48] Daiana N (2021). Improved fermentation strategies in a bioreactor for enhancing poly(3-hydroxybutyrate) (PHB) production by wild type *Cupriavidus necator* from fructose. Heliyon.

[49] Ali I, Jamil N (2014). Enhanced biosynthesis of poly(3-hydroxybutyrate) from potato starch by *Bacillus cereus* strain 64-INS in a laboratory-scale fermenter. Prep. Biochem. Biotechnol..

[50] Gamal RF (2013). Semi-scale production of PHAs from waste frying oil by *Pseudomonas fluorescens* S48. Braz. J. Microbiol..

[51] Gouda MK, Swellam AE, Omar SH (2001). Production of PHB by a *Bacillus megaterium* strain using sugarcane molasses and corn steep liquor as sole carbon and nitrogen sources. Microbiol. Res..

[52] Mitra R, Xu T, Xiang H, Han J (2020). Current developments on polyhydroxyalkanoates synthesis by using halophiles as a promising cell factory. Microb. Cell Fact..

[53] Yamanè, T. & Shimizu, S. Fed-batch techniques in microbial processes. in *Bioprocess Parameter Control* (ed. Fiechter, A.) 147–194 (Springer-Verlag; Berlin/Heidelberg, Germany, 1984).

[54] Bhubalan K (2011). Characterization of the highly active polyhydroxyalkanoate synthase of *Chromobacterium* sp. strain USM2. Appl. Environ. Microbiol..

[55] Chen GQ (2009). A microbial polyhydroxyalkanoates (PHA) based bio and material industry. Chem. Soc. Rev..

[56] Vahabi H (2019). Thermal stability and flammability behavior of poly(3-hydroxybutyrate) (PHB) based composites. Materials (Basel).

[57] Isa MRM (2020). Mechanical, rheological and thermal properties of montmorillonite-modified polyhydroxybutyrate composites. High Perform. Polym..

[58] Frone AN (2019). Morpho-structural, thermal and mechanical properties of PLA/PHB/cellulose biodegradable nanocomposites obtained by compression molding, extrusion, and 3D printing. J. Nanomater..

[59] Budde CF (2011). Growth and polyhydroxybutyrate production by *Ralstonia eutropha* in emulsified plant oil medium. Appl. Microbiol. Biotechnol..

[60] Myers RH, Montgomery DC (2002). Response Surface Methodology: Product and Process Optimization Using Designed Experiments.

[61] Kadier A, Abdeshahian P, Kalil MS, Hamid AA (2018). Optimization of the key medium components and culture conditions for efficient cultivation of *G. sulfurreducens* strain PCA ATCC 51573 using response surface methodology. Iran. J. Sci. Technol. A Trans. Sci..

[62] Braunegg G, Sonnleitner B, Lafferty RM (1978). A rapid gas chromatographic method for the determinationof poly-b-hydroxybutyric acid in microbial biomass. Eur. J. Appl. Microbiol. Biotechnol..

